# News from the Societies

**Published:** 1962-04

**Authors:** 


### *News from the Societies*

### BRITISH MEDICAL ASSOCIATION (Bristol Division)

*Future Programme*

23rd May: at 8.30 p.m.: **Special General Meeting** of Division.

22nd June at 4.30 p.m.: (by invitation). **Tea and Reception for Final M.B., Ch.B.,** Students at Royal Fort.

17th Oct.: at 8.30 p.m.: **Annual General Meeting** of Division.

21st Oct.: at 2.45 p.m.: **St. Luke's Tide Service** at Bristol Cathedral.

6th Nov.: at 8. p.m.: **Annual Dance** at the Berkeley Cafe.

### WESSEX RAHERE CLUB

The Spring Dinner of the above club will take place at Sofroni's Restaurant, Torquay on Saturday, April 14th, under the chairmanship of Mr. Peter Monks of Torquay. It is hoped that a Member of the Staff will be present as Guest of Honour. Further details will be circulated or can be obtained by any Barts. graduates who are not already members, from the Hon Secretary, Mr. A. Daunt Bateman, F.R.C.S., 11 Circus, Bath.

